# Intelligent Framework for Learning Physics with Aikido (Martial Art) and Registered Sensors

**DOI:** 10.3390/s19173681

**Published:** 2019-08-24

**Authors:** Alberto Corbi, Olga C. Santos, Daniel Burgos

**Affiliations:** 1 Research Institute for Innovation & Technology in Education (UNIR iTED), Universidad Internacional de La Rioja (UNIR), 26006 Logroño (La Rioja), Spain; 2 aDeNu Research Group, Artificial Intelligence Department, Computer Science School, Universidad Nacional de Educación a Distancia (UNED), 28040 Madrid, Spain

**Keywords:** inertial sensors, aikido, physics education, moment of inertia, intelligent systems

## Abstract

Physics is considered a tough academic subject by learners. To leverage engagement in the learning of this STEM area, teachers try to come up with creative ideas about the design of their classroom lessons. Sports-related activities can foster intuitive knowledge about physics (gravity, speed, acceleration, etc.). In this context, martial arts also provide a novel way of visualizing these ideas when performing the predefined motions needed to master the associated techniques. The recent availability of cheap monitoring hardware (accelerometers, cameras, etc.) allows an easy tracking of the aforementioned movements, which in the case of aikido, usually involve genuine circular motions. In this paper, we begin by reporting a user study among high-school students showing that the physics concept of moment of inertia can be understood by watching live exhibitions of specific aikido techniques. Based on these findings, we later present *Phy + Aik*, a tool for educators that enables the production of innovative visual educational material consisting of high-quality videos (and live demonstrations) synchronized/tagged with the inertial data collected by sensors and visual tracking devices. We think that a similar approach, where sensors are automatically registered within an intelligent framework, can be explored to teach other difficult-to-learn STEM concepts.

## 1. Introduction

For a long time, there has been a well-acknowledged need to promote the learning of subjects related to science, technology, engineering and mathematics (the so-called STEM areas) to high-school students. The term STEM has recently been extended with an *A* for *arts* [1,2], which may involve humanities, language, dance, drama, music, visual arts, design, new media, and—why not?—also *martial arts*. One of the goals behind STEAM is to teach students innovation, critical thinking and the use of engineering/technology-related concepts/processes in imaginative designs. Other goals of the STEAM ecosystem involve the development of creative approaches and solutions applicable to real-world problems that build on top of the students’ former mathematical/scientific backgrounds [3].

In this paper, we argue that aikido (a defensive martial art based on circular motions and *close-to-elastic* scattering), in combination with simple movement monitoring sensors (i.e., inertial sensors) and visual tracking devices (e.g., simple video cameras, depth recovering gadgets, etc.) can provide a creative way of teaching important concepts related to one of the exponents of the STEM domain: physics. An *intelligent* computing framework capable of registering (both in time and in a 3D Cartesian space) the information of the aforementioned devices is also described in this work. We also assert that this method of transmitting knowledge can be truly understood by high-school students while they remain engaged in the rest of the STEM curricula. In this work, we mainly focus on the concept of *moment of inertia*. This physical quantity (tackled in Section 6.1) is often misunderstood and difficult to *grasp* by young students [4]. The main reason relies on the fact that this concept is addressed from a *demotivating* methodological perspective based on calculations around idealistic geometrical figures (spheres, cylinders, segments rings, etc.) and their associated spinning movements around an isolated axis of rotation. This *otherworldly* learning setting (at least in the physics realm) is felt by students as *unconnected to reality*. In this context, we believe that the human body can also serve as a means of studying the concept of moment of inertia (among others) through a proper affordable multi-sensor and video-enriched framework. Performing movements in aikido involves applying forces, moving fluently through a 3D space (i.e., the *tatami*) and transforming energy. As we look deeper into aikido, we find that this *energy state mutation* should only comprise kinematics. This is well known by aikido masters with a background in science and engineering. However, the benefits of using martial arts (and especially aikido) to understand scientific concepts, and, in particular, those related to physics, have hardly been reported in literature. To the authors’ knowledge, only the works by Mroczkowski, compiled in [5], report a scientifically validated study that shows the value of learning physics and aikido in a combined way. With this motivation, in this paper, we go beyond the aforementioned works and investigate how to support the learning of physics using sensor technology with aikido as a case study, by addressing the following research question:

Can inertial sensors and visual tracking devices enable the production of enriched learning materials that facilitate the understanding of physics ideas by showing, in a combined way, meaningful aikido techniques (performed/explained by martial artists), together with the data provided by these devices?

In order to get our own insights into this matter, we participated in two science outreach related events during the *2019 Education Week* event (presented respectively in Section 3 and Section 5.3). Both experiences have influenced the development of an approach that we have named *Physics + Aikido*, or *Phy + Aik* for short. It consists in an intelligent registering framework to generate innovative educational material. In particular, the goal of *Phy + Aik* is to allow students the access to curated educational material that combines synchronized visual information (high-quality videos and live exhibitions) and tagged data streams collected by inertial sensors. These time series can be produced with the involvement of physics teachers and even real aikido instructors at in situ scenarios or in pre-recorded demonstrations. In addition, *Phy + Aik* can also serve to monitor the movements performed by the students when re-enacting, by themselves, the movements shown in the videos or during the live demonstrations. Such a merging of combined information can eventually contribute to help the student identify real-world forces, movements and related complex magnitudes such as *dynamic momentum*, *angular momentum*, and *moment of inertia*. As it will be further discussed along the present text, the required equipment can be easily acquired and its complexity just lies in the registration process succinctly discussed in Section 5.

Aside from introducing *Phy + Aik*, the rest of the paper is structured as follows. In Section 2, we comment on the background of our approach. In Section 3, we present a user study among high-school students showing that the concept of moment of inertia is better understood by watching live demonstrations of aikido techniques. Then, in Section 4, we introduce the equipment that can be used in *Phy + Aik* to *sensorize* the aikido practice. Next, in Section 5, we present the *Phy + Aik* framework to allow educators generate innovative visual educational material. Following that, in Section 6, we review some physics concepts (including the aforementioned moment of inertia) that could be also be taught with *Phy + Aik*. Finally, some conclusions and ideas for future work are outlined in Section 7.

## 2. Background

As stated in [6], the digital and physical worlds have in recent years opened new possibilities for children and adults to interact with their environment. This interaction is bidirectional: the *environment also interacts with the students*. Such interaction is made mainly possible primarily by 2D bitmap displays and sensors. A sensor is nothing else but *something* (generally an electronic device) that detects or measures a *physical event*. It can also record such episodes/sessions to be reviewed/studied later and it can likewise warn the user in real-time and/or even automatically respond to them. A good example of this last characteristic is the fall detection capabilities of some state-of-the-art *smart watches*, which are able to autonomously call emergency services in case of such an undesirable incident.

As reviewed in [7], inertial sensors (i.e., those that are based on the concept of *inertia*, such accelerometers and gyroscopes) can be used to learn motor skills, which are key in sports and martial arts. The use of sensors in the learning of sports has been widely applied. For instance, in [8], a wearable device is attached to a swimmer in order to improve the learner’s swimming technique. In a very similar way, the authors in [9] evaluate the wrist rotation in golf. In [10], the authors propose a sensor-based system to monitor and assist *snowboarders*. This system goes beyond the typical accelerometer-gyro tandem by adding two pressure sensors in the snowboarder’s shoes. In any case, inertial sensors have proven valuable to measure sports performance, although a particular device may be more suitable and effective than others depending on the sports application [11].

The goal of all of these sensor-based applications is for the wearers to gain (in fact, *learn*) insights about their own motion, style, state, and even the physical environment and conditions in which their practice takes (or took) place. This knowledge is usually gained *offline*, that is, once the exercise has been completed, the learner can review and interpret the recorded data (video sequences and/or inertial records). In addition, the learner can go a step further and *connect* this knowledge to a physical *ground truth* (or theoretical basis). As a result, he/she is able to improve the performance of the physical exercise being tracked (or previously monitored) in combination with the review of the physics processes associated with it. However, in this context, we believe that not only the person carrying out the physical activity, but also external schoolmates reviewing the associated gestures and dynamics, are able to learn the physics involved. As suggested by the outcomes of the study reported in Section 3, this way of learning turns out to be more *useful* than merely studying the theoretical basis in a text book. In this way, it is possible to develop handy learning materials for STEM teaching. This approach is the one followed in *Phy + Aik*, where instead of being based on the practice of more traditional sports activities, it encompasses the application of martial arts (and aikido in particular), which entails additional benefits for learning physics.

## 3. Case Study 

As stated in Section 1, we have carried out a user study to have our own evidence that watching movements of aikido is useful for learning some concepts of physics. Here, we report the user study we have carried out at *Feria Madrid for Science and Innovation 2019*, which was held on 28–31 March 2019 in Madrid (Spain). This scientific event is tailored to high-school students with physics in their curriculum and has the aim of disseminating science in a participatory atmosphere as it is aligned with the *STEMadrid Plan* focused on developing programs that strengthen the interest of teenagers in scientific and technological subjects. This year’s event took place during the *Education Week*. The activity *Physics, Aikido and Artificial Intelligence* was conducted during a four-hour time slot at UNED’s stand on the morning of March 28th, from 10:00 a.m. to 2:00 p.m.

**Figure 1:**
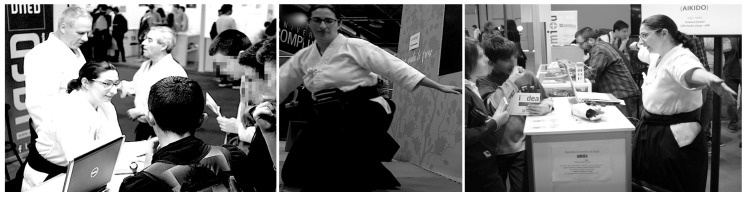
Left: a group of high-school students are asked by the researcher (one of the co-authors of the paper) to participate in the activity. In the background, two members of the *Aikime* dojo. Center: execution of the *suwari waza tai sabaki shikko ho* technique with arms opened (increasing moment of inertia/reducing angular speed). Right: debriefing to the participants after filling out the tests. Photos left and right at *Feria Madrid 2019* event were taken by divulgaUNED and published on Twitter [12].

Young participants who came to the stand (see Figure 1—left) were asked to complete a pre-test covering some concepts of physics and then watched some movements of the aikido practice performed by black belt-level practitioners of the *Aikime* dojo. As detailed below, the concept of moment of inertia was demonstrated with the *shikko* movement performing 180${}^{\circ }$ turns (u-turns) while knee-walking, i.e., performing *suwari waza tai sabaki shikko ho* (or *irimi tenkan shikko*) several times (see Figure 1—center). Then, they were asked to fill out a post-test. After that, the researcher provided some explanations to the participants (Figure 1—right).

The *shikko* exercise can be roughly compared to *walking on knees* (also known as *samurai-walking*). *Shikko* (e.g., Figure 1—center) is very useful for developing awareness of one’s own centre of mass, also known as *hara* in the aikido jargon. This fact contributes to being able to keep a stable position that is later needed for other stand-up techniques. In other words, it can (and effectively does) improve the practitioner’s balance even for movements outside of the *shikko* practice itself. In a previous work [13], we proposed that, through the use of inertial sensors, it is possible to model the aforementioned knee-walking movement by measuring inertial information (acceleration and angular velocity) in the *hara*’s coordinate system. Motion information can be collected with a smartphone attached to the practitioner’s waist using a *fanny pack* (also known as *bum bag*) placed ad hoc or even carefully positioned between the skin (at the level of the navel) and the *aikidogi* (the traditional Japanese garment worn by aikido practitioners). Smartphones are a very useful solution because nowadays they are *ubiquitous devices* (i.e., everyone has one of those), and thus the barrier for their introduction into the classroom for STEAM teaching purposes is lower than using a dedicated device. Of course, as discussed in Section 4, a scientific-range sensor can also be used.

During the execution part of the activity, in some occasions the arms of the aikido practitioners remained widely opened and, in others, they stood close to the body, so that participants could perceive the effect on the speed of the turn due to the change in the body’s contour. The associated physics concept has to do with the fact that the angular momentum (*L*) equals the moment of inertia (*I*) multiplied by the angular velocity (ω). Thus, when the aikido practitioner modifies the shape of his/her body, the moment of inertia changes (i.e., $I⟶I^{\prime }$) because it depends on the distance of each point of the body to the turning axis, so the further the points are from each other (i.e., if the arms are open), the larger the moment of inertia is computed ($I^{\prime }>I$). Since the angular momentum is conserved when no external forces are applied ($L=L^{\prime }$), as in this case, the angular velocity has to inevitably change (i.e., $\omega ⟶\omega^{\prime }$). Specifically, with open arms, the angular velocity is reduced ($\omega >\omega^{\prime }$). Hence, the speed of the turn is *controllable* by opening/closing the arms. All of these concepts were quickly summarized to the students after the live demonstration took place and the post-test was done (Figure 1—right). For the sake of completeness, these ideas can be profoundly tackled (also through motivating software tools) among pre-college students, as suggested in Section 6.1.

**Figure 2:**
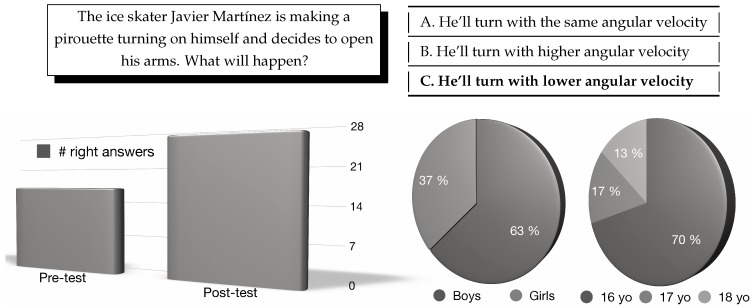
Top: One of the questions asked to the participants as part of the pre- and post-test. The option in bold face is the right one (C). Bottom: Statistical information about the participants in the study: correct answers in pre-test and post-test, gender, and age distribution (*yo* stands for *years old*).

In order to evaluate the understanding of the concept of moment of inertia, the question shown in Figure 2—top, including its three possible answers, was presented to the students who came to the UNED stand, both before (pre-test) and after (post-test) watching the *shikko* movement. A total of 30 students (average age 16.4 years) took part in the study (Figure 2—bottom). In the pre-test, 17 participants answered correctly, while 27 answered correctly in the post-test. The *t*-student analysis shows that the results are statistically significant (t=3.0104; p=0.0054). This field study showed how the u-turn *shikko* movement allowed high-school students to understand the relation between the moment of inertia and angular velocity. In addition, as discussed in the theoretical analysis in Section 6, other concepts besides the moment of inertia can also be learnt through other aikido techniques.

## 4. Monitoring Setup for *Phy + Aik*

We now propose a potential set of affordable sensors and easy to implement methods that can be used to teach physics through genuine aikido techniques, such as *shikko*. These techniques can either be put into practice both in situ (in the context of a conventional physics classroom or as a live demonstration) and as pre-recorded videos/learning material. The main advantage of the approach proposed called *Phy + Aik* is that it can be easily and affordably implemented in schools by teachers and students, and even in collaboration with local aikido clubs.

We also discuss different approaches for using sensors to measure aikido movements. To begin with, modern smartphones already include powerful inertial systems (Section 4.1). However, since smartphones might be difficult to attach to some body parts or to samurai weapons (e.g., to a *bokken*), a second approach could consist in using dedicated integrated devices (such as the scientific-range sensor reviewed in Section 4.2). In addition, stereoscopic cameras can accomplish the derivation of 3D locations from a geometrically registered configuration (Section 4.3). Finally, depth cameras can also allow the achievement of a similar goal but with a single device setup (Section 4.4). A couple of registered depth sensing devices may also allow volume stitching and isosurface derivation.

### 4.1. Smartphone with a Three-Axis Accelerometer + a Three-Axis Gyro

The first proposed device for tracking the motion of aikido practitioners is the set of the factory default inertial sensors (i.e., accelerometer and gyro) packed inside *high-end* smartphones. In particular, we have used three models in our tests: an Apple iPhone 5, an iPhone 8 and a Samsung Galaxy S9 (released in September 2012, September 2017 and March 2018, respectively), although other devices can, of course, suffice. The aforementioned smartphones can be attached to each aikido practitioner with an enclosing fanny pack. A good location might correspond to a place more or less near the *hara* point (discussed in Section 3).

The inertial data measured by such modern smartphones is usually accessible by applications and is even made available to HTML5 APIs [14] and web rendering engines. However, for operational purposes, in the case of iOS-based devices (such as the iPhone 5), we suggest the use of dedicated native applications such as SensorLog, which has allowed us to easily compile the required information. SensorLog (as well as many other Android-based developments) can simultaneously store in disk and stream (via the UDP network protocol) the measured time series via any local WiFi-connected computer (see Section 5.3). All data are timestamped with a millisecond precision. Smartphones have proven to meet the expectations for movement recording, as evinced by [15,16,17].

The acceleration data are given in g units, where a value of 0 g entails *no acceleration* (a *free fall* in the case of the vertical axis). The gyroscopic data has units of angular velocity (rad/s) by default. In the case of the iPhone 5, the accelerometer measures up to ±8 g with a resolution of ±0.002 $m/s^{2}$. The range of the gyro is ±1200 and has a resolution of 0.06 (both in ${}^{\circ }$/s). The data logging in smartphones can usually reach a nominal frequency of 60 Hz, although the subjacent hardware and operative system can lower this rate on some occasions due to battery preservation restrictions. With more detail, the onboard inertial sensors in the iPhone 5 are the L3G4200DH and LIS331DLH three-axis gyro and accelerometer, respectively (both manufactured by STMicroelectronics, Geneva, CH). These same sensors have been also used (in an isolated manner) with success in a variety of studies such as [18,19,20,21,22].

**Figure 3:**
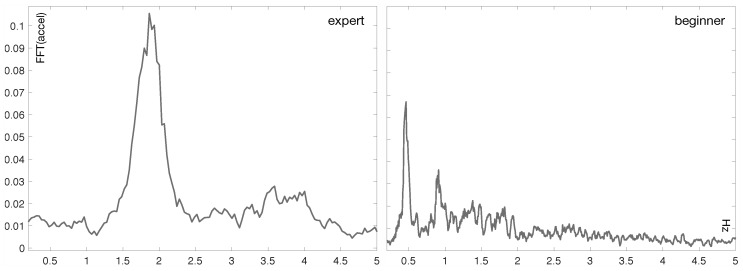
Examples of the accelerometer data of two aikido practitioners with different levels performig the *shikko* movement. The plots show the computed two-sided spectrum of the FFT.

Figure 3 shows two plots of the FFT (Fast Fourier Transform) of the acceleration signal recorded with an iPhone 5 for two aikido practitioners (i.e., an expert and a novice one, respectively) performing the *shikko* movement. The plot corresponding to the expert clearly reveals a central frequency around 2 Hz. On the contrary, the FFT signal related to the newcomer is more heterogeneous.

### 4.2. Scientific-Range Three-Axis Accelerometer Puck

In order to record and replay some specific martial arts-related motions, smaller and more precise sensing devices might be required. For instance, a tiny accelerometer apparatus (such as the AX3) could be mounted on the tip of a wooden sword or *bokken* in order to explain the concept of the linear (also called *tangential*) acceleration, among others.

The AX3 (Axivity, Ltd., Newcastle upon Tyne, UK) is a low cost CE approved logging three-axis accelerometer. The sensor uses a non-volatile flash memory chip and is accessible through a USB-enabled micro-controller. The device is suitable for use in a variety of environments (it is even water resistant up to 1.5 m). The accelerometer has a variable sensitivity to allow it to be used in many applications. The selectable ranges (to balance sensitivity against dynamic range) are: ±2g, ±4g, ±8g, and ±16g, where g is the acceleration due to gravity. Accelerations outside the selected dynamic range result in saturation (*clipping*) of the recorded acceleration. The dynamic range of the accelerometer has no effect on the battery life or memory constraints. This last fact allows us to set the limit of this dynamic range at ±16 g, which is ideal for measuring the three components of the acceleration in fast swing movements.

The AX3 logs time series internally in a binary packed format. This format is named *continuous wave accelerometer* format (CWA) and is very efficient for storing large amounts of data. Its specification is free and there exist implementations for many data processing frameworks, although the use of the default software, OmGui v43 (Axivity, Ltd., Newcastle upon Tyne, UK), is recommended. The AX3 also has a built-in, real-time clock and calendar, which provides the time base for the recorded acceleration data. However, in order to better align/sync the external video stream and the inertial data, we propose the methods described in Section 5. Finally, the AX3 has been used and validated in many scientific studies such as [23,24].

**Figure 4:**
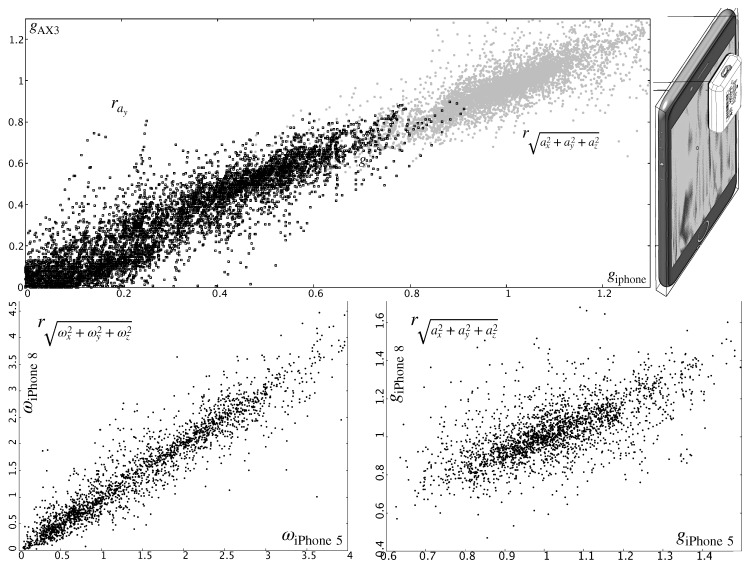
Top: Correlation (for the *y*-component and the vector norm, respectively) between the accelerometer in an iPhone 5 model and a dedicated AX3 sensor (during a 20 m *shikko* track). Bottom: Comparison of the norms of the gyro (ω) and acceleration (*a*) information as measured by two different smartphone models (iPhone 5 and 8) and for the same movement (a similar *shikko* walk).

Even though these types of discrete inertial sensors are, a priori, more accurate than the ones present in consumer-level smartphones, the use of these widespread devices is still totally adequate for the academic purposes described in this work. Figure 4—top shows the correlation in the acceleration measured by an AX3 device and the iPhone 5 smartphone described in Section 4.1. In order to obtain this plot, the AX3 sensor was set aligned relative to the approximate location of the inertial unit. This location can be more or less known or it can be estimated through a (side) didactic experience  [25]. Besides, the quality of the signals among different smartphone models is quite similar, even if they are shipped 3 or 4 commercial seasons apart (as evinced in Figure 4—bottom).

### 4.3. Stereoscopic Cameras

**Figure 5:**
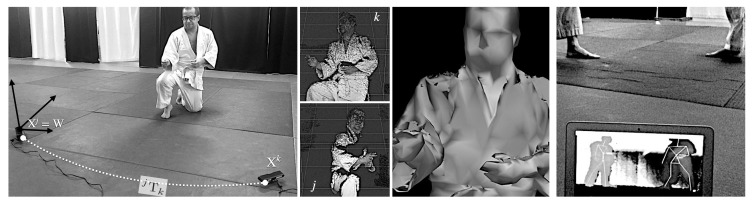
Leftmost images: Two spatially registered depth sensors of ASUS and Microsoft, respectively—corresponding point clouds as measured from both devices and low-quality textured-mesh isosurface reconstruction. Rightmost image: OpenNI-compatible sensor (placed behind the laptop’s screen and not visible in the photographs) that track the movement of two aikido practitioners (*shomen-uchi kotegaeshi*). Their skeletons are derived in real-time and appear in the laptop’s screen. Images correspond to one of the co-authors of the paper and were obtained at *Judelda* dojo (Elda, Spain).

In order to collect motion data, it is also possible to use a pair of webcams in a stereoscopic configuration, as shown in Figure 5—left. Each camera defines its own geometrical setting (*j*, *k*), and the coordinates of their focal point is labeled with $X^{j}$ and $X^{k}$, respectively. This combined optical information can be used to three-dimensionally track, for instance, the face of the aikido practitioner (as seen from each video system), and, therefore, infer his/her location in the room. From this position (and in combination with time), it is also possible to extract other physical parameters, such as speed or acceleration. This step is also part of the intelligent registration system described in Section 5.2.

Other means of optically tracking moving bodies, such as dedicated fiducial markers, can be used. However, we suggest the exploitation of the simplest and most direct fiducial marker possible: a person’s own face. A fiducial system also allows the derivation of the *pose* of each camera (location and orientation relative to a fixed point in the scene). These types of fiducial systems are different from the ones tackled in Section 5.1, whose goal there is to synchronize (in time) the video and the motion data acquired by other devices. Face-tracking algorithms are nowadays very easy to implement and can smoothly run even in web-based environments. Two good examples are [26,27]. An example of our efforts in this area is commented in Section 6.4.

**Figure 6:**
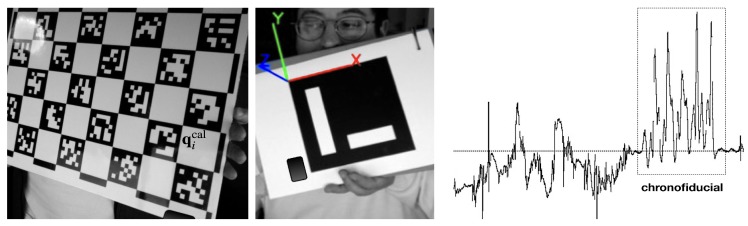
Summary of the method for multi-camera and inertial sensor registration. The (acceleration) signal on the right plot shows a pre-arranged number of spikes that are generated by gently waving the calibration board a prearranged number of times. During this operation, the board appears blurry and the intelligent framework (inevitably) stops identifying fiducials. The spiky pattern in the inertial signal (and the moment when it occurred) can be automatically detected. The time series is recorded by a rigidly attached accelerometer (in this case, an AX3 device glued to the board pictured at the left). The small tracking board at the center also contains a glued AX3 accelerometer on its surface.

The stereoscopic camera system is directly wired to a simple integrated computer that accommodates the dual video data. In order to combine several images of the same practitioner at each geometrical setting, we need the associated camera projection matrices $P^{j}$, $P^{k}$. These matrices are calculated in advance through a quick calibration phase with, for instance, a classical chessboard pattern [28], as shown in Figure 6—left. These calibration frames are also equipped with fiducials $Q_{i}$ that are then projected (when photographed) to $q_{i}$ spots in the image. In the case of a chessboard panel, $Q_{1}$, $Q_{2}$, etc., are the intersections of the white and black squares (which can be automatically identified). Combinations of $Q_{i}$, $q_{i}$ pairs are then fed into the *direct linear transform* or DLT [29] and projection matrices can then be derived. With $P^{j}$ and $P^{k}$, we can finally relate 3D points ${}^{W}Q_{i}$ (expressed in an external coordinate reference frame called *world* or W), with their 2D *observed* projections $q_{i}^{j}$, $q_{i}^{k}$ on each image pair (*j* and *k*). For simplicity, we can define W to be coincident with $X^{j}$, that is: ${}^{X^{j}}T_{W}=\left[I\right]$, where I is the identity transformation matrix. Using projective geometry, we can write:

(1) $${\hat{q}}_{i}^{j}=P^{j}\cdot^{W}{\hat{Q}}_{i}\;\;{\hat{q}}_{i}^{k}=P^{k}\cdot^{W}{\hat{Q}}_{i},$$

where ${}^{W}{\hat{Q}}_{i}$, and ${\hat{q}}_{i}^{j}$, ${\hat{q}}_{i}^{k}$ are the homogeneous coordinates of ${}^{W}Q_{i}$ and $q_{i}^{j}$, $q_{i}^{k}$, respectively. With an RQ decomposition, $P^{j}$ and $P^{k}$ can be expressed as:

(2) $$P^{j}\approx K^{j}\;\;P^{k}=K^{k}\cdot {}^{X^{k}}T_{W},$$

where $K^{j}$ and $K^{k}$ are two 3×3 upper triangular matrices that contain the intrinsic parameters of each camera system (for a given geometrical setting *j* or *k*) and ${}^{X^{k}}T_{W}$ is a rigid transformation that translates 3D homogeneous points relative to W (coincident with $X^{j}$) to the coordinates of $X^{k}$. In other words, ${}^{X^{k}}T_{W}$ geometrically connects the *focal point* of both cameras. In addition, if both devices are from the same manufacturer (and same model), the intrinsic parameters in K are therefore the same and it is only necessary to calculate them once (i.e., $K^{j}=K^{k}=K$). Sometimes, intrinsics can be easily derived from the camera specifications (e.g., sensor width and height and optical focal length).

The process of deriving 3D info from pairs of registered images is also known as *projection-to-volume* reconstruction or *projective reconstruction* and is described in Section 12.2 of [30]. It enables the determination of the 3D location of an observed point $q_{i}$ in two images (*j* and *k*). Given two projection matrices $P^{j}$ and $P^{k}$ and using Equation (1), we can write:

(3) $${\hat{q}}_{i}^{j}=K^{j}\cdot {\hat{Q}}_{i}\;{\hat{q}}_{i}^{k}=P^{k}\cdot {\hat{Q}}_{i},$$

where ${\hat{q}}_{i}^{j}$ and ${\hat{q}}_{i}^{k}$ represent the ground truth. Since we are working with homogeneous coordinates, the equivalence between two points has to be expressed using the cross product:

(4) $$\begin{array}{c} {\hat{q}}_{i}^{j}\times {\hat{q}}_{i}^{j}={\hat{q}}_{i}^{j}\times K^{j}\cdot^{w}{\hat{Q}}_{i}=0\;\;\;{\hat{q}}_{i}^{k}\times {\hat{q}}_{i}^{k}={\hat{q}}_{i}^{k}\times P^{k}\cdot^{w}{\hat{Q}}_{i}=0. \end{array}$$

Each of these expressions determines two linearly independent equations that can be written in the form of a linear system. When solved through a single value decomposition method (SVD), we can derive the 3D location of a specific point ${\hat{Q}}_{i}$ observed in the two images (Figure 5—left/center). All these mathematical operations involve simple matrix operations that students at high-school levels can comfortably carry out (and/or the teacher can explain their calculation with open source tools [31]).

### 4.4. Depth Cameras

Modern depth cameras rely on the acquisition of *point clouds*. These data structures can be obtained with devices such as the reputed Microsoft Kinect™ or other OpenNI-compatible sensors [32], like the ones shown in Figure 5—right. A point cloud is an organized or unorganized set of points [Q] in a 3D space whose coordinates are usually expressed relative to the W frame.

Modern consumer depth cameras use *structured light* and machine learning. Inferring the position of a body entails: (1) computing a depth map (using structured light) and (2) inferring the position of the body by means of machine learning. The depth map is built by analyzing a speckle pattern of infrared laser light. This entails the projection of a known pattern onto the scene and the determination of its depth from the deformation of the aforementioned ornaments. There exist several machine learning implementations for skeleton tracking. One of the most widely used and deployed versions (and also embedded in the software inside many of the consumer sensors) is NiTE, succinctly described in [33]. These kinds of sensors have also been used to monitor and infer movements from practitioners in other martial arts, such as Taekwondo [34]. Kinect-like devices not only measure depth, but, as affirmed above, they can also report a body’s position and track movements with fair precision, even for specific body parts and quick movements. This feature is shown in Figure 5—right to track the movement of two aikido practitioners when performing a movement called *shomen-uchi kotegaeshi*.

If two depth cameras are registered (as explained in Section 5.2), it might be possible to achieve a minimal stitching of both point clouds and derive the corresponding isosurface (Figure 5—center). This meshed compound can be refined thanks to hole-filling and smoothing algorithms present in widely used free software packages, such as MeshLab, Blender or Paraview. The generation of this volume turns out to be very useful to estimate the moment of inertia, as discussed in Section 6.1.

## 5. Generating Learning Material with *Phy + Aik*

We now describe how our approach *Phy + Aik* can generate synchronized multimedia educational material tagged with inertial data that can be useful in teaching some important physics concepts, such as the moment of inertia, its relationship with the angular velocity and the conservation of the angular momentum (which is especially relevant in the *shikko* movement). This is obtained with the generation of high-quality videos and visual compositions that simultaneously display aikido movements and the corresponding stream of inertial data. Through simple movement monitoring sensors and visual tracking devices (e.g., simple video cameras, depth recovering gadgets, etc.), it is possible to provide a creative way of teaching STEM concepts that can be truly understood by high-school engaged students.

One of the strengths of *Phy + Aik* is grounded on the fact that the number and type of the interplayed sensors can grow at will while maintaining a gentle profile of affordability and ease of use. In fact, *Phy + Aik* can start with a single smartphone (as mentioned in Section 4.1), which is nowadays a ubiquitous low-price device. Nevertheless, as the amount and variety of sensors grows (such as those described in Section 4), it becomes necessary the design of an *intelligent framework* that alleviates and automates as much as possible the necessary registration/calibration phase. This step becomes even more critical when video data are added to the *information mix*. However, before describing our full (and automatic) approach to this technical obstacle (Section 5.2), we quickly review next a semi-automatic scenario. This setting may turn out to be more appropriate (and less complex) for the case of having just an inertial sensor and a video stream. In addition, in Section 5.3, we comment on the possibility of carrying out live demonstrations to present visual educational materials to the students.

### 5.1. Manual Video and Time Series Data Alignment

Data alignment can be achieved with, for instance, ELAN (*EUDICO Linguistic Annotator*). ELAN is a tool that allows the creation, edition, visualization and search of annotations within video and audio data. It was also originally developed with the aim to provide a technological basis for the annotation and exploitation of multi-media recordings. Certainly, it is specifically designed for the analysis of language and gestures, but it can be used for other purposes. For instance, thanks to its annotating capabilities, it is possible to sync (i.e., align in time) video and streams of inertial data. In fact, a key aspect when capturing experimental data is to spatially (and chronologically) register several streams of data and ELAN excels at this feature. Even though these streams are sometimes time-tagged in advance, they may have insufficient resolution or be out of phase with one another. For this reason, in order to achieve such registration, it is necessary to place visual markers in all of the involved streams. Such markers can retrospectively be used to identify certain event starts and stops.

A good *time marker* or *chronofiducial* should have the following characteristics: be tolerant to the orientation of the devices, be easily identifiable in the data set, occur over a short period of time, and be repeatable. With this approach, video and time series alignment is carried out manually. In the next section, we propose an automatic/intelligent framework that not only simplifies this positioning operation, but also ensures that all video/depth capturing devices and inertial sensors are registered both in a space and time coordinate frame.

### 5.2. Intelligent Framework for Multi-Sensor and Video Registration

In this section, we propose an intelligent framework for multi-sensor and video registration. The goal of an automatic registration (or calibration) phase is to unattendedly locate a set of spatial and chronofiducials (introduced respectively in Section 4.3 and Section 5.1) in the streams provided by different sensors. The registration step may also deliver the intrinsic parameters of the available cameras (RGB, depth, etc.) and its geometrical relation with other contour recognition equipment. These transformations remain invariant during the execution of the aikido practice (such as the *shikko* movement) and/or as long as the multi-imaging system moves rigidly.

A simple chessboard-like registration frame (shown in Figure 6—left) was designed to help during this phase. To be more specific, the chessboard pattern is not the classical one based on equally spaced black and white squares, but on the system devised by [35]. The advantage of this fiducial set (and the associated algorithm and C++ implementation) is that it is very robust and fast, even under occlusion events. The board also contains a rigidly attached inertial sensor. This sensor can be a smartphone or even a discrete sensor (such as the one mentioned in Section 4.2) and can be placed on the front face of the registration board or on its back. With this marker set, we can derive the camera equations and the geometrical relation between the conventional cameras and other present contour sensors. In the case of OpenNI-based depth cameras, integrated video and depth streams are usually pre-registered as a factory default setting, which turns out very helpful in the registration phase: the reported 3D data are already expressed (*pre-transformed*) relative to the RGB optical center. The registration process proposed, which is very similar to the one described in [36,37], can be summarized as follows:

1. The registration frame is introduced in the scene and placed as still as possible (this is the only manual step). Video (and depth, if applicable) streams are continuously recorded. It is assumed that each system manages its own real-time clock and that those streams are a priori out-of-sync. Modern video and multimedia containers (Quicktime, Matroska, etc.) usually have encoded a creation_date metadata item that accounts for the date and time in which the recording started [38], according to the computer managing the video capture. The teacher just has to make sure that the pattern in the registration frame is correctly visible by every camera (and/or the video counterpart of all the present depth cameras) without interruption.
2. Using the *direct linear transform* (DLT) algorithm [30], the 3D location $Q_{i}^{\mathrm{cal}}$ of each marker in the board (corners) and the coordinates of the corresponding 2D projections $q_{i}^{\mathrm{cal}}$ in the images, we obtain the registration matrix for each device. It is assumed that optical distortions and intrinsic parameters have been previously derived or are known (device manufacturer specifications). This process is automatic and can run in real time in modern (but still modest/low price) hardware. Preliminary results can even be shown as graphical overlays.
3. Using the video streams of the registration frame (and its detected visual markers thanks to the OpenCV-based algorithm presented in [35]), it is possible to obtain the rigid transformations that link the reference frames of all devices. In this way, all cameras can now be virtually positioned relative to a common (spatial) coordinate system.
4. The board is then shaken *N* times. During this phase, the board appears *blurry* from the point of view of all video/depth devices and fiducials are no longer detected (corners also appear fuzzy). However, the attached inertial sensor does effectively *sense* and measure this (also *timestamped*) wavy time series pattern. In this context, the rules regarding the generation of chronofiducials (listed at the end of Section 5.1) are fully respected.
5. An intelligent peak detection process is executed following the algorithm described in [39] in order to find the series of *N*-spikes in the acceleration stream of a chosen Cartesian axis (depending on the position/orientation of the inertial sensor in the registration board and the specific selected direction of agitation). The chronofiducial (needed for the time alignment) is established as the moment in which the first agitation/peak in the signal took place.
6. The initial *pose* (relative orientation) and location of all inertial sensors (if there are more present than the one stuck to the registration board) is determined by attaching a corresponding coplanar visible fiducial, as in the example shown in Figure 6—center.
7. Likewise, these sets of sensors and visible markers are shaken the same number of *N* occasions. The intelligent peak detection commented in step 5 is also run for those inertial series.
8. All time series/stamps are finally aligned (in time) and, as stated in step 3, the geometrical transformations of all sensors are derived relative to the W frame (i.e., one of the video cameras).

With this approach, even with humble hardware and modest computing setups, it is possible to achieve an unnoticeable time lag in the registration of all video/inertial signals. It is also feasible to achieve a spatial resolution of 1 cm. All in all, the resolutions/precisions delivered by this automatic intelligent framework are adequate for the academic purposes described in the scope of *Phy + Aik*.

It is very important from a pedagogical, psychological and perceptual point of view to have the correct synchronization of all data streams. To our knowledge, this approach has not been proposed previously. The inertial data can be stored for later production of high-quality videos or real-time streamed to projectors or big screens in auditorium rooms. This comes very much handy when using *Phy + Aik* approach during presentations (and also during live exhibitions), as discussed next.

### 5.3. Real-Time Stream of Data

Live demonstrations are also a key component in education even if that implies that the student apparently remains as a passive viewer of a screen with data. This concept has been coined as *interactive screen experiment* [40]. In *Phy + Aik*, we have also had into account these scenarios in which aikido techniques take place live and direct in front of an audience of motivated learners. In these environments, students can witness (or even perform by themselves in a tutored way) the execution of these aikido movements while they can simultaneously read the inertial signals (captured by the worn sensors) in big monitors and external screens. In this context, the broadcasting and live data plot can be achieved mainly by combining UDP-stream of inertial data and screen sharing. Both approaches were tested also during the 2019 Education Week, but in another event called AULA 2019, in the context of the activity *Aikido and Artificial Intelligence* organized on March 29th (between 15:00 and 17:00 h) in the UNED stand, and also with the participation of black-belts of the *Aikime* dojo. During the live demonstration at AULA 2019 the aikido practitioners wore a smartphone attached to their waists on the *hara* point and performed several techniques in front of the audience, including the *shikko* movement as shown in Figure 1—center, to show in real-time and on a big screen the change of the inertial signals in relation to the movements being performed.

## 6. Physics Concepts

We now review some physics concepts that can be addressed in a *Phy + Aik*-enabled physics classroom. As noted in previous sections, with *Phy + Aik* the learning experience around the physics subject is complemented with stimulating educational material grounded on the practice (or the viewing) of aikido techniques enhanced with tagged inertial information and accompanied by proper live or prerecorded explanations handled by aikido masters and/or physics teachers.

### 6.1. Moment of Inertia and Angular Momentum

**Figure 7:**
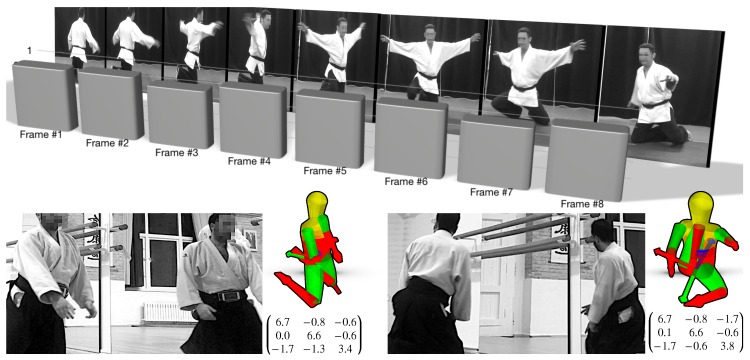
Top: Example of the constancy of the angular momentum (normalized $L_{zz}$ component, specifically) in the case of a high moment of inertia *shikko* u-turn (open arms). Bottom: Approximate reconstruction of the tensor of inertia with the Yeadon package. This last manual task can be facilitated if the movement is performed in front of a mirror (which allows the viewing from other angles, as in this example). Photos taken at *Judelda* dojo (Elda, Spain) and *Sintagma* dojo (Valencia, Spain), respectively.

The concept of moment of inertia and its study within the context of *Phy + Aik* has already been mentioned along the present article. As stated in Section 1, this notion is seldom compared to the mass of an object and is almost always presented to the student from an uninspiring point of view. Connected to it, the angular momentum is the *rotational equivalent* of the linear version. As with the moment of inertia, it is also seldom compared with Newton’s second law of dynamics and it is an important quantity in nature because it is conserved. Together with mass, charge and spin (among others), the fact of the preservation of the values of these physical quantities is itself a very important postulate in any STEM study, and in physics in particular. Thanks to *Phy + Aik*, this conservation can be visually shown during the knee u-turnings in the *shikko* movement. The practitioner can force/simulate a change in his/her moment of inertia by, for instance, extending or contracting the length of his/her arms while performing a spin between the forward and backward trajectories. In particular, Figure 7—bottom shows the normalized $L_{zz}$ component calculated (as explained below) for some steps during an open-arms *shikko* u-turn. Although there are some minor differences (<5%), the values can be considered more or less equal.

The fact of calculating the angular momentum necessarily involves obtaining the moment of inertia of the body performing (or *suffering*) the rotational movement. As stated in Section 1, these estimations (as executed in scholar environments) do not involve meaningful connections to real applications. However, in the context of *Phy + Aik*, students can calculate the very close-to-real moment of inertia of a human body (an aikido practitioner performing a *shikko* u-turn, for instance). The students can also learn that the moment of inertia is not a scalar, but something more complex called *tensor*. In order to calculate this tensor, the teacher may allow the students to work with the Yeadon package. This software (written in Python), calculates the masses, center of mass positions, and inertia tensors following the human inertia model proposed in [41]. Yeadon provides an easy and intuitive 3D visualization interface based on the MayaVi package which outputs the final moment of inertia. Through this GUI, the students can gently adjust the position of joints, upper limbs, etc., in order to mimic as faithfully as possible the physical configuration involving an aikido movement (e.g., *shikko*). For this process, the students can watch the registered video streams. If these videos are recorded in front of a mirror, this might help to see the same scene from different points of view. Within *Phy + Aik*, it is also possible to use two registered RGB cameras (as discussed later in Section 6.4) or two registered depth cameras (as previously discussed in Section 4.4). In this last case, it is not only viable to watch two 2D images from two points of view, but also to *navigate* through two 3D point clouds. Finally, if the transformation between both point clouds is known, a 3D meshed isosurface may be reconstructed (Figure 5—center), which might help the student to configure the human model in Yeadon. In the case of pre-college students, it is possible to only pay attention to the most significant term of the tensors. In the case of *shikko*, this term is $I_{zz}$, which is equivalent to the most relevant component around the vertical or *craniocaudal* axis.

### 6.2. Integration and Dead Reckoning

**Figure 8:**
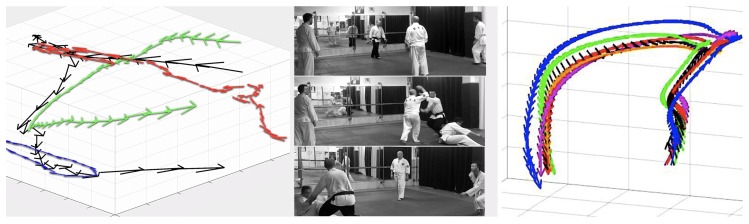
Left and middle images: Dead reckoning application from the measured acceleration streams obtained during a multiple attacker freestyle exercise (*taninzu gake*). The arrow plot on the left shows the trajectories (and speed at each point) of each *uke* or attacker (blue, green and red colors) and the *tori* or defender (black color) in the 3D space (shown as photographs on the right). The size of the arrows represents the intensity of the speed of each partner. Photos taken at *Judelda* dojo (Elda, Spain). Rightmost image: Derived tracks of the dead reckoning between four AX3s running at 3200 (black), 1600 (green), 800 (red) and 100 (blue) Hz, respectively, and two iPhone models: 6S+ (orange track) and 5 (purple track) running both at 100 Hz. The orientation/size of the arrows reflects the velocity vector at each given position. The difference in the mean position (between all devices) is obvious, but not significant for the learning scenarios described in this text. In the case of the speed, the mean difference is just a few cm/s (also negligible for the academic purposes regarding the learning of physics).

The definition of dead reckoning is very well established in [42,43]. It basically states that it is possible to derive an object’s *followed path* from the double integration of the vector components of the instant acceleration. Of course, a small drift may still take place and it heavily depends on the sampling resolution in the time and frequency domains when digitizing the acceleration signals. As an example of this drift, Figure 8 shows the paths derived from the data measured by four AX3 accelerometers and two different iPhone models worn near the *hara* point by four different aikido practitioners and running at different sampling frequencies during a multiple attacker freestyle exercise.This accumulated error can, however, be assumable for small displacements and if the data pulling frequency is on the order of the tens of Hertz (as it is our case, according to the operational performance discussed in Section 4.1).

Certainly, the concept of integration (of the acceleration signal in the time domain) is somewhat complex for high-school students [44]. However, a real/useful example of dead reckoning might contribute to understanding the reasoning behind this mathematical operation. Dead reckoning can also be achieved by dividing the FFT-transformed acceleration by the scale factor represented by the square of the frequency and taking the inverse FFT of it. This last method is well described in [45] and allows for working on other valuable mathematical/physical concepts such as the *Nyquist frequency*.

With the application of dead reckoning shown in Figure 8—left it is not only possible to derive the position from accelerometer data, but also the instantaneous speed. A position-speed phase diagram may also reveal the circular/spiral motions genuine to the martial art of aikido.

### 6.3. Spherical and Cylindrical Coordinates

The term *coordinate system* appears in the STEM curriculum linked to physics. All of the inertial sensors described and used within *Phy + Aik* collect data in a Cartesian coordinate frame. This presents a nice opportunity to introduce this concept to the students. It also entails an appropriate occasion to present the theory behind the transformations between coordinate systems and to introduce (possibly for the first time) other types of reference frames for 3D representation, such as spherical and cylindrical coordinates. In addition, as stated throughout this text, the aikido martial art is closely attached to circular motions. This can be shown in a clearer way through the use of sword-like movements represented in the spherical and cylindrical systems. For instance, the work described in [46] uses a cylindrical-like reference frame for representing *bokken* swings.

### 6.4. Basic Optics and Epipolar Geometry

The concepts of epipolar geometry, image projection and stereo (and eventually, 3D volume) reconstruction are relatively complex and require a decent level of knowledge about geometry and matrix calculus. However, as discussed in Section 6.2, these ideas can be presented more intuitively by inviting the learner to reflect on the geometrical relation between the *real* 3D world and the 2D image. It is also a pertinent moment to familiarize the students with the notion of *pinhole cameras* [47].

**Figure 9:**
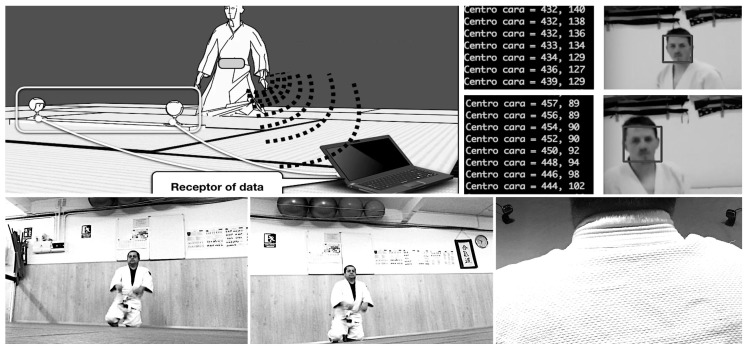
Top-left: Setup of the dual camera system and face detection for stereoscopic reconstruction (designed for the *MyShikko* prototype [13]). A smartphone is schematically represented (attached to the practitioner’s *hara* and wirelessly transmitting inertial data to a host), but it is not necessary for the stereo 3D reconstruction. Top-right: The face recognition algorithm is being applied on the captured video from an aikido practitioner while walking on knees. The numerical output in the consoles represents (in time) the 2D coordinates of the center of the face being detected. Epipolar geometry later allows the derivation of 3D spots (tackled in Section 4.3 and Section 6.4). Bottom: Registered cameras (shown at the right image) in a stereoscopic configuration. Photos taken at *Judelda* dojo (Elda, Spain).

The mathematical background of the 2D-to-3D back projection is summarized in Section 4.3. Through simple (and familiar) diagrams, students can achieve some insight about how two cameras *see* the same point in the space and why it is necessary to *pair* at least two of them in order to reverse the 3D-to-2D projection operation. While one of the cameras projects a 3D point as a compact set of pixels, the other casts an infinite line (the so-called *epipole*). Thanks to a stereo camera configuration, it is possible to track someone’s position with fixed points in two (or more) images (for instance, an aikido practitioner’s face, like in Figure 9—top-right), as initially discussed in [13].

### 6.5. Other Concepts

Many other notions connected to physics can be tackled within the context of *Phy + Aik*. For instance, the use of Kinect-like devices allows the introduction of *infrared light* and the *electromagnetic spectrum*. Additionally, the inherent circular/spiral movements proper to aikido enable the analogy to other crucial processes, ideas and modern experiments, such as those related to *particle physics*, *detectors*, *nonlinear behavior*, *chaos dynamics*, *attractors*, etc. Students can also learn the importance of registration and simultaneity of events (commented in Section 5) and why they are of key relevance in modern physics experiments. Young learners usually take for granted that the succession of two events does not require any extra step for its correct matching and identification. Nevertheless, the study of anything that takes place in the *fabric of space-time* always needs the definition of a pre-agreed reference frame in which objects (such as aikido practitioners) and actions are determined relative to it.

In addition, it should be noted that (as already commented in Section 4.3, Section 6.1 and Section 6.4) most physics-related concepts can be taught to students even if they do not have the appropriate mathematical background. This can be achieved by succinctly explaining the calculations with appropriate software tools, simplifying the mathematical operations or using intuitive graphics and animations that invite the learner to reflect on the relation between some variables/processes. This is also an example of how, in line with the STEM culture, scientific, technological, engineering and mathematical ideas can be taught in an interrelated way.

## 7. Conclusions and Future Work

In this paper, we have presented *Phy + Aik*, a STEAM-based approach whose goal is to introduce a range of physics-related ideas to high-school students through curated educational materials that combine aikido techniques with the data gathered by a variety of inertial and optical sensors. To test our approach, we carried out a field study with high-school students that confirmed that the concept of moment of inertia is better understood by watching live demonstrations of aikido techniques. *Phy + Aik* interplays the inertial information gathered by motion sensors and the input of video/depth recording devices into a registered common space-time reference frame. These data aggregation and stream registration can take place in a semi-manual manner or in an almost automated way thanks to the intelligent registering framework also described in the text. In this context, inertial sensors, cameras and depth sensors are arranged into an organized four-dimensional space coordinate system through the use of markers (both in the space and time dimensions). These fiducials are, in turn, automatically detected. With *Phy + Aik*, educators can either produce innovative visual educational lectures consisting of high-quality videos (synchronously tagged with the inertial data collected by motion sensors), or organize live demonstrations in collaboration with aikido practitioners/masters.

Our future research is framed in the INT^2^AFF project (*INTelligent INTra-subject development approach to improve actions in AFFect-aware adaptive educational systems*), where we plan to explore the affective life cycle when learning STEM concepts with the support of martial arts. In any case, *Phy + Aik* is a first step toward the design of intelligent psychomotor tutoring systems, where students’ movements are used as input data in the tutoring system so that personalized STEM contents based on students’ motion information can be recommended, as discussed elsewhere [7]. For this, motion information obtained with inertial sensors can be modeled with artificial intelligence techniques [48].

## References

[1] Land M.H. (2013). Full STEAM ahead: The benefits of integrating the arts into STEM. Procedia Comput. Sci..

[2] Maeda J. (2013). STEM + Art = STEAM. STEAM J..

[3] Jolly A. STEM vs. STEAM: Do the Arts Belong?. https://www.edweek.org/tm/articles/2014/11/18/ctq-jolly-stem-vs-steam.html.

[4] Rimoldini L.G., Singh C. (2005). Student understanding of rotational and rolling motion concepts. Phys. Rev. Spec. Top. Phys. Educ. Res..

[5] Mroczkowski A. (2012). Using the knowledge of Biomechanics in teaching Aikido. Injury and Skeletal Biomechanics.

[6] Schneider J., Börner D., Van Rosmalen P., Specht M. (2015). Augmenting the Senses: A Review on Sensor-Based Learning Support. Sensors.

[7] Santos O.C. (2016). Training the Body: The Potential of AIED to Support Personalized Motor Skills Learning. Int. J. Artif. Intell. Educ..

[8] James D.A., Davey N., Rice T. An accelerometer based sensor platform for insitu elite athlete performance analysis. Proceedings of the Sensors, 2004 IEEE.

[9] Ghasemzadeh H., Loseu V., Jafari R. (2009). Wearable coach for sport training: A quantitative model to evaluate wrist-rotation in golf. J. Ambient Intell. Smart Environ..

[10] Spelmezan D., Schanowski A., Borchers J. Wearable automatic feedback devices for physical activities. Proceedings of the Fourth International Conference on Body Area Networks.

[11] Espinosa H.G., Shepherd J.B., Thiel D.V., Worsey M.T.O. (2019). Anytime, Anywhere! Inertial Sensors Monitor Sports Performance. IEEE Potentials.

[12] https://twitter.com/divulgaUNED/status/1111370950375862273.

[13] Corbí A., Santos O.C. MyShikko: Modelling knee walking in Aikido practice. Proceedings of the Adjunct Publication of the 26th Conference on User Modeling, Adaptation and Personalization.

[14] Puder A., Tillmann N., Moskal M. Exposing native device APIs to web apps. Proceedings of the 1st International Conference on Mobile Software Engineering and Systems.

[15] Kos A., Tomažič S., Umek A. (2016). Suitability of smartphone inertial sensors for real-time biofeedback applications. Sensors.

[16] Kos A., Tomažič S., Umek A. (2016). Evaluation of smartphone inertial sensor performance for cross-platform mobile applications. Sensors.

[17] Del Rosario M.B., Redmond S.J., Lovell N.H. (2015). Tracking the Evolution of Smartphone Sensing for Monitoring Human Movement. Sensors.

[18] Mourcou Q., Fleury A., Franco C., Klopcic F., Vuillerme N. (2015). Performance evaluation of smartphone inertial sensors measurement for range of motion. Sensors.

[19] D’Alessandro A., D’Anna G. (2013). Suitability of low-cost three-axis MEMS accelerometers in strong-motion seismology: Tests on the LIS331DLH (iPhone) accelerometer. Bull. Seismol. Soc. Am..

[20] Zhao X., Han R., Yu Y., Hu W., Jiao D., Mao X., Li M., Ou J. (2016). Smartphone-based mobile testing technique for quick bridge cable-force measurement. J. Bridge Eng..

[21] Yao X., Sun G., Lin W.Y., Chou W.C. The design of a real-time accelerometer-based sleeping position monitoring system and its application on obstructive sleep apnea syndrome. Proceedings of the International Conference on Systems and Informatics (ICSAI).

[22] Schirmer G. (2013). Software and System Development of a Smartphone-Based Flight-Tracking Device to Enhance Flight Instruction. Ph.D. Thesis.

[23] Khan A.M., Kalkbrenner G., Lawo M. Recognizing physical training exercises using the Axivity device. Proceedings of the ICT Meets Medicine and Health.

[24] Clarke C.L., Taylor J., Crighton L.J., Goodbrand J.A., McMurdo M.E., Witham M.D. (2017). Validation of the AX3 triaxial accelerometer in older functionally impaired people. Aging Clin. Exp. Res..

[25] Mau S., Insulla F., Pickens E.E., Ding Z., Dudley S.C. (2016). Locating a smartphone’s accelerometer. Phys. Teach..

[26] Lundgren E., Rocha T., Rocha Z., Carvalho P., Bello M. tracking.js: A Modern Approach for Computer Vision on the Web. https://trackingjs.com.

[27] Papoutsaki A., Sangkloy P., Laskey J., Daskalova N., Huang J., Hays J. Webgazer: Scalable webcam eye tracking using user interactions. Proceedings of the International Joint Conference on Artificial Intelligence (IJCAI).

[28] Bradski G., Kaehler A. (2008). Learning OpenCV.

[29] Aziz A.Y.I., Karara H.M. Direct linear transformation into object space coordinates in close-range photogrammetry. Proceedings of the Symposium on Close-Range Photogrammetry.

[30] Hartley R., Zisserman A. (2004). Multiple View Geometry in Computer Vision.

[31] Eaton J.W., Bateman D., Hauberg S. (2002). GNU Octave Manual.

[32] Falahati S. (2013). OpenNI Cookbook.

[33] Ramos E. (2012). Kinect Basics. Arduino and Kinect Projects.

[34] Choi C.H., Joo H.J. (2016). Motion recognition technology based remote Taekwondo Poomsae evaluation system. Multimed. Tools Appl..

[35] Garrido-Jurado S., Muñoz-Salinas R., Madrid-Cuevas F.J., Marín-Jiménez M.J. (2014). Automatic generation and detection of highly reliable fiducial markers under occlusion. Pattern Recognit..

[36] Albiol F., Corbi A., Albiol A. (2016). Geometrical Calibration of X-Ray Imaging with RGB Cameras for 3D Reconstruction. IEEE Trans. Med. Imaging.

[37] Corbi A., Albiol F., Albiol A. Joint calibration of RGB + X-ray cameras. Proceedings of the 2016 Global Medical Engineering Physics Exchanges/Pan American Health Care Exchanges (GMEPE/PAHCE).

[38] Gilmer B. (2004). File Interchange Handbook for Images, Audio, and Metadata.

[39] Scholkmann F., Boss J., Wolf M. (2012). An efficient algorithm for automatic peak detection in noisy periodic and quasi-periodic signals. Algorithms.

[40] Kirstein J., Nordmeier V. (2007). Multimedia representation of experiments in physics. Eur. J. Phys..

[41] Yeadon M.R. (1990). The simulation of aerial movement—II. A mathematical inertia model of the human body. J. Biomech..

[42] Kok M., Hol J.D., Schön T.B. (2017). Using inertial sensors for position and orientation estimation. arXiv.

[43] Park S.K., Suh Y.S. (2010). A zero velocity detection algorithm using inertial sensors for pedestrian navigation systems. Sensors.

[44] Knight J.F., Bristow H.W., Anastopoulou S., Baber C., Schwirtz A., Arvanitis T.N. (2007). Uses of accelerometer data collected from a wearable system. Pers. Ubiquitous Comput..

[45] Han S. (2010). Measuring displacement signal with an accelerometer. J. Mech. Sci. Technol..

[46] James D., Uroda W., Gibson T. Dynamics of swing: A study of classical Japanese swordsmanship using accelerometers. Proceedings of the 2nd Asia-Pacific Congress on Sports Technology.

[47] Mihas P., Andreadis P. (2005). A historical approach to the teaching of the linear propagation of light, shadows and pinhole cameras. Sci. Educ..

[48] Santos O.C. (2019). Artificial Intelligence in Psychomotor Learning: Modeling Human Motion from Inertial Sensor Data. Int. J. Artif. Intell. Tools.

